# A Si/SiC Heterojunction Double-Trench MOSFET with Improved Conduction Characteristics

**DOI:** 10.3390/mi16121335

**Published:** 2025-11-27

**Authors:** Yi Kang, Dong Liu, Tianci Li, Zhaofeng Qiu, Shan Lu, Xiarong Hu

**Affiliations:** 1 School of Electrical Engineering, Southwest Jiaotong University, Chengdu 611756, China; yikang@my.swjtu.edu.cn (Y.K.); 2024210806@my.swjtu.edu.cn (T.L.); zfq2002@my.swjtu.edu.cn (Z.Q.); mountainlu@my.swjtu.edu.cn (S.L.); 2 School of Integrated Science and Engineering, Southwest Jiaotong University, Chengdu 611756, China; 3 School of Science, Xihua University, Chengdu 610039, China; hxr2013@mail.xhu.edu.cn

**Keywords:** Si/SiC heterojunction, heterojunction tunneling, double trench MOSFET, conduction characteristics, bipolar degradation

## Abstract

A Si/SiC heterojunction double-trench MOSFET with improved conduction characteristics is proposed. By replacing the N+ source and P-ch regions with silicon, the device forms a Si/SiC heterojunction that exhibits Schottky-like characteristics, effectively deactivating the parasitic PiN body diode and improving third-quadrant performance. A high-k gate dielectric is incorporated to induce a strong electron accumulation layer at the heterointerface, thinning the energy barrier and enabling tunneling-dominated current transport, thereby significantly enhancing the first-quadrant performance. TCAD simulation results demonstrate that the proposed device achieves a specific on-resistance (*R*_on,sp_) of 1.78 mΩ·cm^2^, representing a 20.5% reduction compared to the conventional SiC DTMOS, while maintaining a comparable breakdown voltage (BV) of approximately 1380 V. A significant reduction in the third-quadrant turn-on voltage (*V*_on_) is achieved with the proposed structure, from 2.74 V to 1.53 V. Meanwhile, the unipolar conduction mechanism similar to that of Schottky effectively suppresses bipolar degradation. To enhance device reliability, the design incorporates a trenched source and heavily doped P-well, which collectively mitigate high electric field concentrations at the trench corners. The proposed device offers an integration strategy enhancing both forward conduction and reverse conduction in high-voltage power electronics.

## 1. Introduction

Silicon carbide (SiC) MOSFETs are highly competitive for medium- and high-voltage power electronics due to the superior material properties of SiC, including its wide bandgap, high critical breakdown field, and excellent thermal conductivity [1,2,3]. These characteristics make it a promising candidate for next-generation power switching devices.

However, SiC MOSFETs still face several critical technological challenges, particularly at the SiO_2_/SiC interface, where high interface state density causes strong carrier scattering [4,5,6,7,8]. Despite extensive research, the channel mobility in SiC MOSFETs remains approximately one order of magnitude lower than that of silicon-based devices and far below the bulk mobility of SiC [5,9,10]. This low mobility directly results in high channel resistance. To overcome this limitation, the Si/SiC VDMOS heterostructure has been introduced [11]. It leverages the superior channel mobility of silicon to effectively reduce *R*_on,sp_ [12]. Recent investigations have further developed this concept. Chen, H. et al. demonstrated a Si/4H-SiC VDMOS incorporating an electron tunneling layer, which substantially lowers on-state resistance by facilitating electron tunneling through a narrowed heterojunction barrier [13]. Wang et al. proposed a triple RESURF Si/SiC heterojunction LDMOS complete with an analytical model [14]. The practical implementation of these heterojunction devices relies heavily on advanced wafer bonding techniques for creating high-quality Si/SiC interfaces. Two primary techniques, epitaxial growth and wafer bonding, have been employed for fabricating Si/SiC heterostructures [15,16,17]. Wafer bonding approaches include bonding 4H-SiC to silicon-on-insulator substrates and direct silicon-to-SiC bonding [18]. The surface activation bonding (SAB) method enables heterostructure formation at room temperature, and post-bonding annealing can further enhance the interface quality [19]. Liang, J. et al. utilized this technique to fabricate heterojunction bipolar transistors, while Gammon, P.M. et al. developed Si/SiC configurations for lateral power devices using similar processes [20,21]. These methods successfully overcame lattice mismatch challenges, producing interfaces with strong mechanical integrity and thereby advancing the feasibility of Si/SiC integration. Recent research has not only guaranteed reliable electrical interfaces but also improved thermal boundary conductance, which is a crucial factor for power device applications [22,23].

SiC MOSFETs commonly use their parasitic body diode as a freewheeling path in converters. However, this diode suffers from a high *V*_on_ and susceptibility to bipolar degradation, which limits system performance [24,25]. Integrated unipolar diodes, such as junction barrier Schottky (JBS), Schottky barrier diodes (SBD), and merged PiN/Schottky (MPS) diodes, have been developed to improve reverse conduction [26,27,28,29]. Nonetheless, these solutions consume additional chip area and worsen the trade-off between *V*_on_ and *R*_on,sp_. Another approach involves MOS-channel diode (MCD) designs, such as DioMOS, MCD-MOSFET, and embedded low-barrier diode MOSFETs [30,31,32]. However, they may compromise reliability without careful design of the oxide and doping profiles. In this context, heterojunction diodes present a promising alternative for future device architectures [33]. The Si/SiC heterojunction provides a superior alternative with its lower potential barrier compared to SiC pn junctions, enabling reduced *V*_on_ while avoiding bipolar degradation [34].

In this work, a Si/SiC heterojunction double-trench MOSFET, named HJ-DTMOS, is proposed and demonstrated via TCAD simulations. In the proposed HJ-DTMOS, the conventional N+ source and P-base regions of a 4H-SiC double-trench MOSFET are replaced with Si, forming a Si/SiC heterojunction. This heterojunction exhibits Schottky-like behavior, which deactivates the parasitic PiN diode. A high-k gate dielectric is employed to form an electron accumulation layer at the heterointerface, reducing the barrier thickness and enabling tunneling-dominated current transport, thereby lowering the *R*_on,sp_. Additionally, a trench source terminal and heavily doped P-well are incorporated to mitigate high oxide electric fields at the trench bottom and corners. Consequently, without compromising the BV, the HJ-DTMOS exhibits significant performance improvements in both the first and third quadrants.

## 2. Device Structure and Mechanism

**Figure 1:**
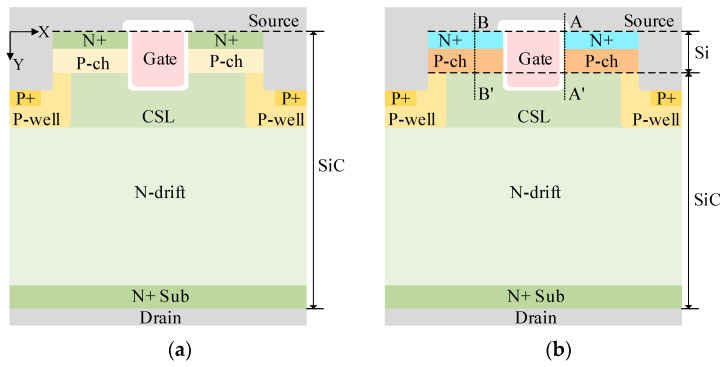
Schematic cross-section of (**a**) Con-DTMOS and (**b**) HJ-DTMOS.

Figure 1a,b shows the schematic cross-sections of the conventional SiC DTMOS (Con-DTMOS) and HJ-DTMOS, respectively. The key feature of HJ-DTMOS is the replacement of the N+ source and P-ch regions with silicon. By forming a heterojunction diode (HJD) between the Si P-ch and the SiC current spreading layer (CSL) that behaves similarly to a Schottky barrier diode (SBD), the parasitic PiN diode operation is suppressed due to its lower *V*_on_. Additionally, the use of a high-k gate dielectric promotes a high electron accumulation layer, offering a low-barrier conduction path for electrons [35]. The N-drift region is 10 μm thick with a doping concentration of 5 × 10^15^ cm^−3^ to support blocking voltages exceeding 1200 V. The trench source and heavily doped P-well are employed to mitigate the high oxide electric field at the trench bottom and corners. The incorporation of CSL offers a compromise between oxide reliability and low *R*_on,sp_ by improving current distribution. Key design parameters for both structures are summarized in Table 1. The parameters for the Con-DTMOS are based on the structure reported in [36].

Sentaurus TCAD simulations are used to analyze the performance of the HJ-DTMOS and the Con-DTMOS, considering Shockley–Read–Hall recombination, Auger recombination, Okuto–Crowell impact ionization, doping-dependent transport, enormal, bandgap narrowing, incomplete dopant ionization, and material anisotropy [37]. In addition, the barrier-lowering and tunneling model is used for simulating the behavior of HJD. The active area is set as 1 cm^2^ for all cases.

**Figure 2:**
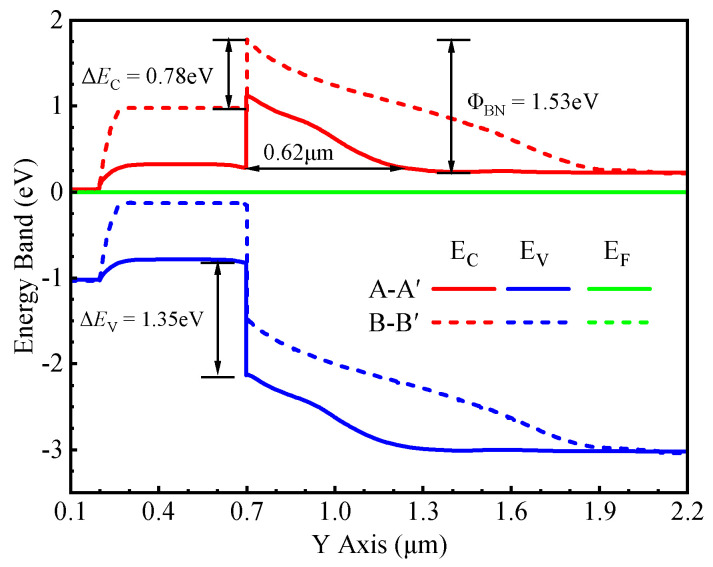
Energy band diagram at thermal equilibrium along A–A′ and B–B′ cut-lines.

The energy band diagram of the Si/SiC heterojunction under thermal equilibrium along the paths [A–A′ and B–B′ lines in Figure 1b] is shown in Figure 2. The heterojunction has a conduction band barrier gap (∆*E_C_*) of 0.78 eV and a valence band barrier energy gap (∆*E_V_*) of 1.35 eV. This high electron barrier height (Φ_BN_) of approximately 1.53 eV results in a low *V*_on_ for the HJD. It is also noteworthy that at *V*_gs_ = 0 V, the high-k dielectric induces an accumulation of electrons at the gate oxide interface, effectively creating an inversion layer. This phenomenon transforms the heterojunction near the gate oxide from a p–N type (comprising p-type narrow-gap and N-type wide-gap semiconductors) into an n–N type (comprising n-type narrow-gap and N-type wide-gap semiconductors).

**Figure 3:**
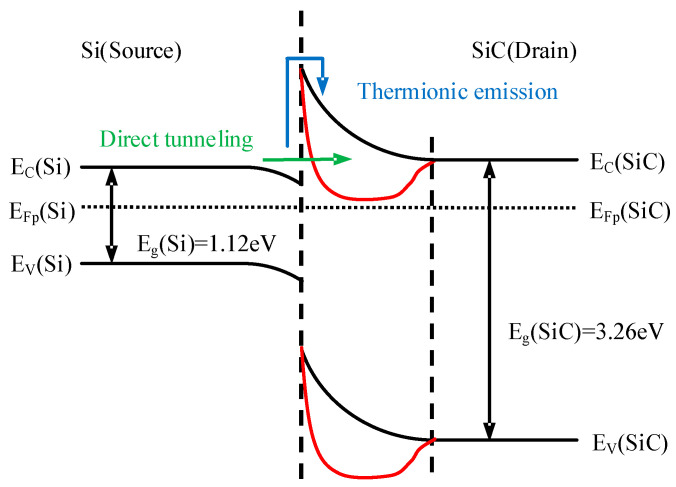
Energy band diagram of the n–Si/n–SiC heterojunction.

Figure 3 (black line) shows the energy band diagram at the n–Si/n–SiC heterojunction interface. Two distinct electron transport mechanisms exist at this interface: thermionic emission and direct tunneling, represented schematically by the blue and green paths in Figure 3, respectively. Under zero external bias, electron transport from the Si to the SiC side is governed by thermionic emission. In this regime, electrons must possess sufficient kinetic energy to overcome the heterojunction barrier, resulting in only a small fraction of the electron population being able to surmount the HJD barrier. However, as the barrier width narrows (as shown by the red curve in Figure 3), a finite probability for direct tunneling emerges. The tunneling probability is critically dependent on the distance the electron must tunnel through; a shorter distance yields an exponentially higher probability. When the barrier width is reduced to a sufficiently small value, the tunnel current increases dramatically, causing direct tunneling to become the dominant transport mechanism. This transition enables a substantially higher overall current.

**Figure 4:**
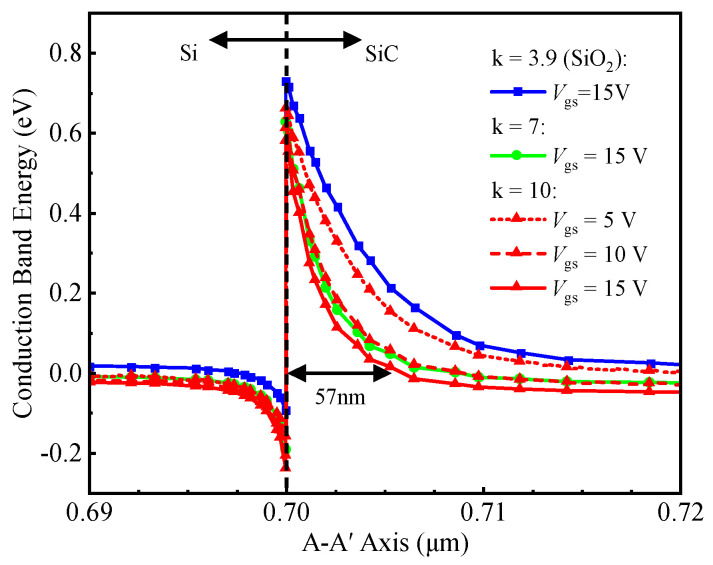
Conduction band energy along the tunneling path at different k-values and *V*_gs_.

Figure 4 shows the conduction band energy along the A–A’ cutline [in Figure 1b] under varying k-values and *V*_gs_. At *V*_gs_ = *V*_ds_ = 0 V, the 0.62 μm depletion layer (in Figure 2) sustains drain voltage by blocking electron flow from Si P-ch to SiC CSL, enabled by heterojunction and P-well/CSL junction effects. Within effective tunneling distance, electrons tunnel through the heterojunction barrier into the SiC conduction band. Increasing *V*_gs_ enhances electron accumulation near the gate trench, thinning the heterojunction barrier. This enhanced accumulation reduces the width of the heterojunction barrier, thereby facilitating electron tunneling. At *V*_gs_ = 15 V and k = 10, the barrier thickness decreases to 57 nm, enabling efficient electron tunneling from silicon to SiC and forming a forward tunneling current.

**Figure 5:**
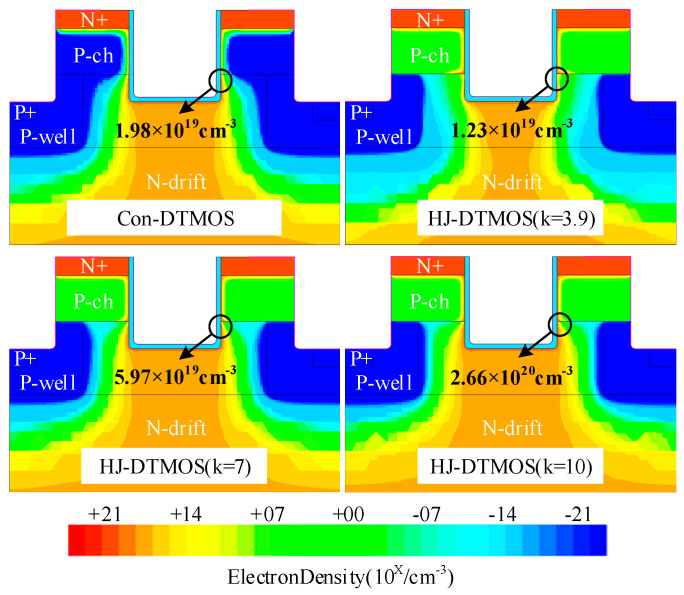
Electron density distribution of Con-DTMOS and HJ-DTMOS (k = 3.9, 7, 10) at *V*_gs_ = 15 V and *V*_ds_ = 5 V.

Figure 5 compares the electron density distribution of Con-DTMOS and HJ-DTMOS (k = 3.9, 7, 10) at *V*_gs_ = 15 V and *V*_ds_ = 5 V. The heterojunction in HJ-DTMOS (k = 3.9) reduces electron density from 1.98 × 10^19^ cm^−3^ to 1.23 × 10^19^ cm^−3^ compared to Con-DTMOS due to depletion effects. High-k dielectrics enhance lateral electric fields, promoting electron accumulation at the semiconductor–dielectric interface. This accumulation intensifies with increasing k, peaking at 2.66 × 10^20^ cm^−3^ for k = 10, approximately ten times that of the Con-DTMOS. It is also noteworthy that, due to the spatially limited influence of k and *V*_gs_, the width of the electron tunneling region is constrained. As a result, the HJ-DTMOS exhibits a higher electron density in the P-ch region compared to the Con-DTMOS, since electrons that fail to tunnel successfully accumulate within the P-ch region.

HJ-DTMOS employs a high-k dielectric to generate a dynamically tunable electron accumulation layer. This approach enables real-time modulation of the barrier width through gate bias, facilitating a balance between on-state conduction and off-state blocking capabilities. Consequently, it offers design flexibility compared to static doping-based methods [13].

**Figure 6:**
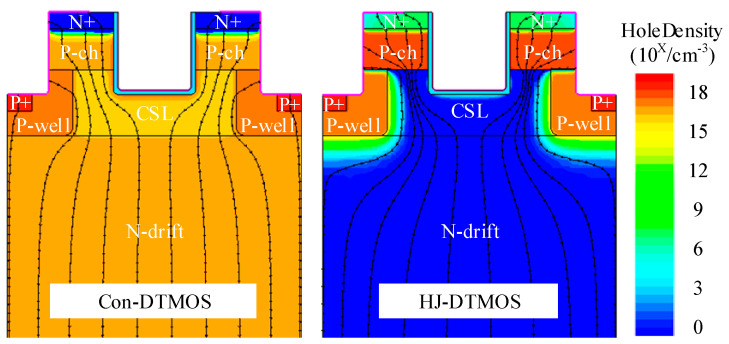
Hole density and total current flowlines distribution for Con-DTMOS and HJ-DTMOS (k = 10) at *I*_ds_ = −100 A/cm^2^.

Figure 6 compares the hole density and total current flowline distribution for Con-DTMOS and HJ-DTMOS at *I*_ds_ = −100 A/cm^2^. The Con-DTMOS operates in bipolar conduction mode, where current flows primarily through the parasitic PiN diode. In contrast, the HJ-DTMOS exhibits significantly reduced hole density within the N-drift region. This suppression of hole transport arises from the large valence band offset at the HJD, which impedes hole movement toward the drain while permitting electron current flow toward the source. Consequently, the markedly lower hole density in the HJ-DTMOS drift region confirms its unipolar conduction mechanism, which is similar to that of SBDs and distinct from the bipolar operation observed in Con-DTMOS.

## 3. Simulation Results and Discussion

The electrical characteristics of the HJ-DTMOS are compared with those of Con-DTMOS. The comparison includes *R*_on,sp_, *V*th, *V*_on_, and *BV*. The effects of the high-k dielectric and interface charge density are also analyzed.

**Figure 7:**
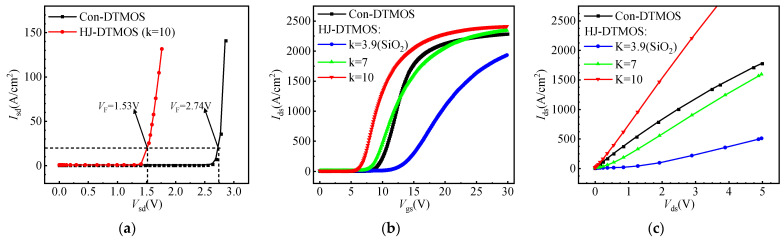
(**a**) Reverse conduction I–V characteristics of the Con-DTMOS and HJ-DTMOS under Vgs = –5 V. (**b**) Transfer characteristics and (**c**) output I–V characteristics of the Con-DTMOS and HJ-DTMOS at various k-values.

As shown in Figure 7a, the Con-DTMOS exhibits high *V*_on_ due to dominant conduction through its SiC PiN body diode. Conversely, HJ-DTMOS uses unipolar electron transport through its low Φ_BN_ HJD, reducing *V*_on_ to 1.53 V from 2.74 V at *I*_sd_ = 20 A/cm^2^. Figure 7b presents transfer characteristics of Con-DTMOS versus HJ-DTMOS with varying k-values. The transfer characteristics reveal that a higher k-value significantly improves gate control. The HJ-DTMOS with k = 3.9 shows a higher *V*th and poorer gate control than the Con-DTMOS due to inadequate barrier thinning. In contrast, the k = 10 device exhibits a markedly left-shifted curve, demonstrating enhanced gate modulation that reduces *V*th substantially from 10.8 V to 5.8 V.

Figure 7c compares conduction I-V characteristics of Con-DTMOS and HJ-DTMOS with varying k-values. At low *V*_gs_, HJ-DTMOS with small k exhibits nonlinear *I*_ds_-*V*_ds_ behavior due to insufficient electron accumulation for heterojunction barrier thinning, preventing direct tunneling. Higher k-values compensate for this limitation through enhanced electron accumulation. At *V*_gs_ = 15 V, HJ-DTMOS displays ideal resistive IV curves, similar to the Con-DTMOS. Specifically, at *V*_gs_ = 15 V, HJ-DTMOS (k = 10) achieves a 20.5% lower *R*_on,sp_ of 1.78 mΩ·cm^2^ compared to the 2.24 mΩ·cm^2^ of Con-DTMOS. This reduction is attributed to the significantly lower resistance of the Si channel compared to that of the SiC channel. Although the heterojunction introduces additional heterointerface resistance [38], the combined resistance of the heterojunction and the Si channel under gate bias remains lower than that of the conventional SiC channel.

**Figure 8:**
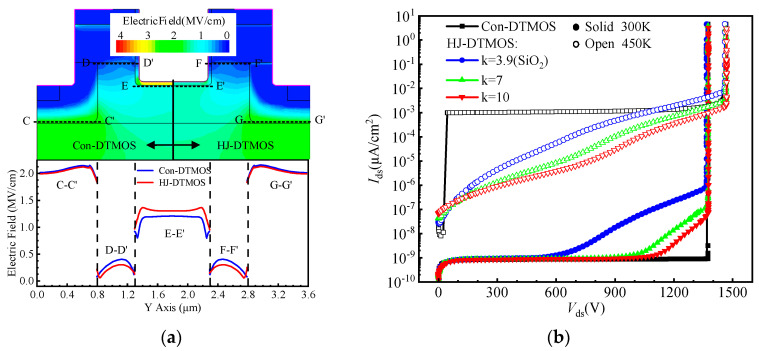
(**a**) Distribution of electric field along the P-well/N-drift junctions (C–C′ and G–G′), the heterojunctions (D–D′ and F–F′), and the trench bottom (E–E′) of Con-DTMOS and HJ-DTMOS (k = 10) at *V*_ds_ = 1200 V. (**b**) Blocking characteristics of the Con-DTMOS and the HJ-DTMOS.

Figure 8a illustrates the electric field distribution of Con-DTMOS and HJ-DTMOS at *V*_ds_ = 1200 V. By redistributing the electric field, the high-k dielectric reduces the field across the gate oxide and augments it in the underlying 4H-SiC. This augmented field, whose magnitude correlates positively with the k-value, intensifies the JFET effect between the 4H-SiC region and the adjacent P-well, consequently alleviating the field at the heterojunction interface. Therefore, a higher k-value accordingly results in a stronger suppression of the electric field at the heterojunction interface.

Figure 8b shows the blocking characteristics of the Con-DTMOS and the HJ-DTMOS at room temperature and high temperature. At room temperature, both the Con-DTMOS and HJ-DTMOS exhibit highly similar electric field distributions, with the peak field located at the P-well corner, as revealed in Figure 8a. This similarity enables both devices to maintain a comparable avalanche breakdown voltage of approximately 1380 V. However, differences in the bulk and surface electric fields lead to distinct leakage currents. Owing to its heterojunction properties, the HJ-DTMOS exhibits a leakage current that is orders of magnitude higher than that of the Con-DTMOS at high voltages. Notably, a higher k-value effectively suppresses the electric field at the heterojunction interface, which consequently reduces the leakage current. At elevated temperatures, however, thermionic emission becomes the dominant leakage mechanism across the heterojunction, strongly dependent on barrier height and temperature. Consequently, the leakage current of HJ-DTMOS increases with rising temperature. Rising temperature increases the breakdown voltage of HJ-DTMOS primarily due to enhanced lattice scattering, which impedes carrier acceleration and necessitates a higher voltage to initiate avalanche multiplication.

**Figure 9:**
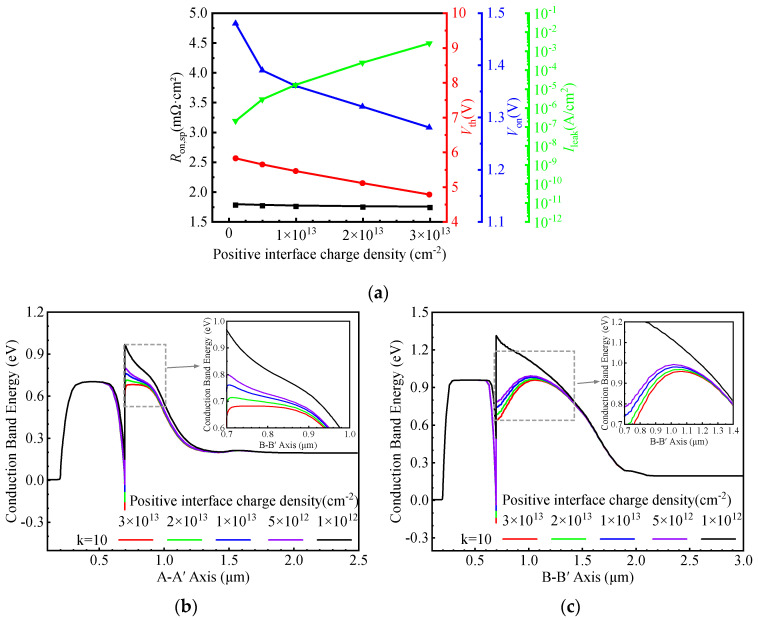
(**a**) Dependence of *R*_on,sp_, *V*th, *V*_on_, and *I*_leak_ on the positive interface charge density. Energy band diagrams at thermal equilibrium along the (**b**) A–A′ and (**c**) B–B′ cut-lines for different positive interface charge densities.

Figure 9a illustrates the influence of positive interface charge density on the electrical characteristics of the HJ-DTMOS. The band modulation at the Si/SiC interface is driven by an internal electric field induced by interface charges, and its intensity increases with the charge density.

Figure 9b presents the equilibrium energy band diagrams along the A–A′ cutline under various positive interface charge densities. A higher density of positive interface charges leads to more significant downward band bending at the interface, facilitating the formation of an electron accumulation layer. Under forward conduction, the heterojunction barrier width is reduced, promoting electron tunneling and improving carrier transport. As a result, both *R*_on,sp,_ and *V*th decrease with elevated positive charge density.

Figure 9c shows the corresponding band diagrams along the B–B′ cutline. As the positive charge density increases, the dominant factor determining Φ_BN_ shifts from the heterointerface toward the SiC bulk, leading to a gradual reduction in *V*_on_. The BV remains largely unaffected by variations in interface charge density, owing to the shielding effect of the dual P-well structure. However, at 1200 V, the leakage current (*I*_leak_) increases sharply with higher positive charge density, resulting in long-term reliability degradation. Therefore, to ensure stable device operation, the positive interface charge density should be minimized.

**Figure 10:**
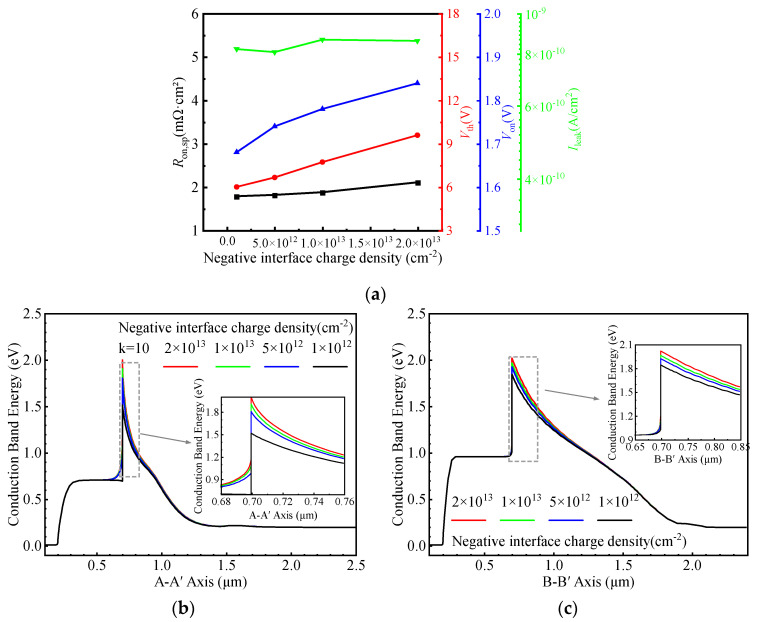
(**a**) Dependence of *R*_on,sp_, *V*th, *V*_on_, and *I*_leak_ on the negative interface charge density. Energy band diagrams at thermal equilibrium along the (**b**) A–A′ and (**c**) B–B′ cut-lines for different negative interface charge densities.

Figure 10a illustrates the influence of negative interface charge density on the electrical characteristics of the HJ-DTMOS. Figure 10b presents the equilibrium energy band diagrams along the A–A′ cutline under various negative charge densities. In contrast to positive interface charge, negative interface charges cause significant upward band bending at the interface, thereby reducing the local electron concentration. Under forward conduction conditions, the heterojunction barrier width increases, which hinders electron tunneling. Consequently, both *R*_on,sp,_ and Vth increase with rising negative charge density. Figure 10c shows the corresponding energy band diagrams along the B–B’ cutline. As the negative charge density increases, the Φ_BN_ continually rises, leading to a gradual increase in *V*_on_. Although the *I*_leak_ exhibits some fluctuation with increasing negative charge density at 1200 V, the BV remains largely unaffected. Therefore, to ensure satisfactory conduction performance of the device, the negative interface charge density should be minimized.

**Figure 11:**
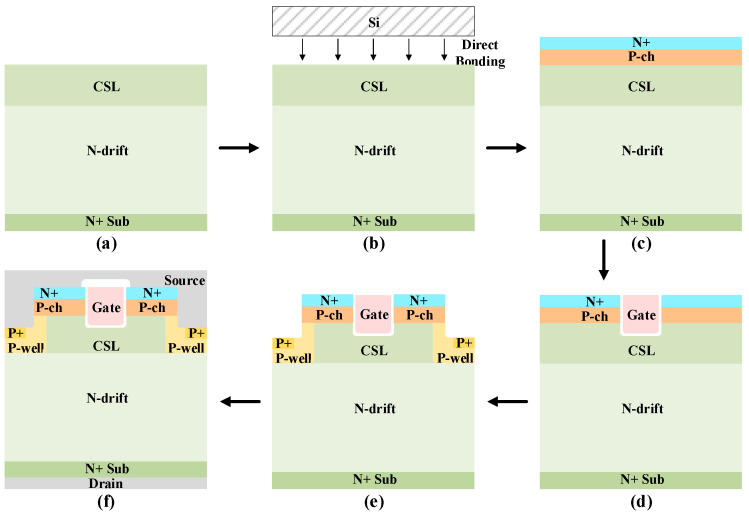
Key fabrication process steps for the HJ-DTMOS: (**a**) Form the epitaxial N-drift layer and CSL on N+ substrate; (**b**) Form Si/SiC heterostructures by direct bonding; (**c**) Form the P-ch region and N+ source region by double implantation; (**d**) Etch the gate trench, deposit a high-k gate dielectric by ALD, and deposit polysilicon for the gate; (**e**) Etch source trench and ion implantation to form the P-well region and P+ region; and (**f**) Form the source and drain electrodes.

Figure 11 shows a possible process for the proposed HJ-DTMOS. First, the N-drift region is formed by epitaxial growth on the N+ substrate, as shown in Figure 11a. Then, the CSL region is formed by epitaxial growth. A high-quality Si/SiC heterostructure is subsequently realized using surface activation bonding (SAB), accompanied by a controlled post-bonding annealing step, as shown in Figure 11b [23]. The P-ch and the N+ source regions are then fabricated through double implantation, depicted in Figure 11c. Thereafter, a gate trench is etched, and a high-k dielectric gate oxide is deposited via atomic layer deposition (ALD) [39]. The gate electrode is subsequently deposited and patterned, as presented in Figure 11d. A source trench is then formed, and the P-well and P+ regions are defined by tilted ion implantation along its sidewall, as shown in Figure 11e. Finally, a passivation layer is deposited, contact windows are etched, and ohmic contacts are established, completing the structure in Figure 11f.

## 4. Conclusions

This work proposed and thoroughly investigated, through TCAD simulations, a Si/SiC heterojunction double-trench MOSFET (HJ-DTMOS). The key feature of this structure is the strategic replacement of the conventional SiC N+ source and P-channel regions with silicon, forming a Si/SiC heterojunction. This design effectively addresses two fundamental limitations of standard SiC MOSFETs: low channel mobility and bipolar degradation of the body diode. Simulation results demonstrate significant performance improvements: the *V*_on_ is reduced to 1.53 V (from 2.74 V in conventional devices), effectively suppressing bipolar degradation, while the *R*_on,sp_ is lowered by 20.5% to 1.78 mΩ·cm^2^ without compromising the breakdown voltage. These enhancements are attributed to the heterojunction’s Schottky-like behavior and the high-k dielectric-induced tunneling transport. While the trenched source and heavily doped P-well enhance reliability by mitigating electric field concentrations, the quality of the interface remains critical for achieving optimal performance. This work demonstrates the HJ-DTMOS’s strong potential for high-performance power applications, with experimental validation planned for future research.

## Figures and Tables

**Table 1:** Device parameters for simulations.

Parameters	HJ-DTMOS	Con-DTMOS
Cell pitch (μm)	3.6	3.6
Si layer thickness(μm)	0.7	-
SiC layer thickness(μm)	12.8	13.5
N-drift thickness (μm)	10	10
N-drift doping (cm^−3^)	5 × 10^15^	5 × 10^15^
CSL thickness (μm)	0.8	0.8
CSL doping (cm^−3^)	1 × 10^16^	1 × 10^16^
P-ch thickness (μm)	0.5	0.5
P-ch doping (cm^−3^)	2 × 10^17^	2 × 10^17^
Trench depth (μm)	1	1
Trench width (μm)	1	1
Gate oxide thickness (nm)	50	50

## Data Availability

The original contributions presented in this study are included in the article. Further inquiries can be directed to the corresponding author.

## Abbreviations

The following abbreviations are used in this manuscript:

HJ-DTMOS	Si/SiC heterojunction double-trench MOSFET
Con-DTMOS	Conventional double-trench MOSFET
HJD	Heterojunction diode
SBD	Schottky barrier diode
JBS	Junction barrier Schottky diode
MPS	Merged PiN/Schottky diode
MCD	MOS-channel diode
DioMOS	Diode-integrated MOSFET
CSL	Current spreading layer
SAB	Surface activation bonding
ALD	Atomic layer deposition
SRH	Shockley–Read–Hall recombination
ΔE_c_	Conduction-band offset at Si/SiC heterojunction
ΔE_v_	Valence-band offset at Si/SiC heterojunction
Φ_BN_	Electron barrier height of heterojunction
k	Relative permittivity of gate dielectric
*R*_on,sp_	Specific on-resistance
BV	Breakdown voltage
*V*_on_	Turn-on voltage
*V*_ds_	Drain-to-source voltage
*I*_ds_	Drain-to-source current
*I*_sd_	Source-to-drain current
*V*_gs_	Gate-to-source voltage
*V*th	Threshold voltage
*I*_leak_	Leakage current
A–A′, B–B′	Cut-line labels in figures
C–C′, D–D′, E–E′, F–F′, G–G′	Electric-field profile cut-lines
